# Identification of a *Penicillium oxalicum* fungus isolate and its pathogenicity against *Panonychus citri* (McGregor)

**DOI:** 10.3389/fmicb.2025.1619976

**Published:** 2025-08-06

**Authors:** Danchao Du, Jia Lyu, Zhengdong Huang, Baoju An, Li Zhu, Zhanxu Pu, Shunmin Liu, Xiurong Hu, Lianming Lu

**Affiliations:** ^1^The Citrus Research Institute of Zhejiang Province, Taizhou, China; ^2^Key Laboratory of Fruit and Vegetable Function and Health Research of Taizhou, Taizhou, China

**Keywords:** *Panonychus citri*, biological control, *Penicillium oxalicum*, spore, entomopathogen

## Abstract

To obtain efficient biocontrol fungi against *Panonychus citri*, this study systematically identified the highly pathogenic strain HYC2101 through an analysis of its cultural characteristics, physiological and biochemical properties, and molecular identification. The isolate was identified as *Penicillium oxalicum*. In laboratory observations using stereomicroscopy and scanning electron microscopy, the infection process of *P. oxalicum* HYC2101 in *P. citri* was documented, and its pathogenicity against female adults and larvae was determined. The optimal temperature range for the mycelial growth and conidial production of *P. oxalicum* HYC2101 was found to be 25–35°C, with the highest sporulation on SDAY (sabouraud dextrose yeast extract agar medium). Observations of the infection process revealed that conidia easily attached to the cuticular folds and setae of the mite. After 24 h, the spores germinated and penetrated the cuticle. By 48 h, the hyphae had invaded the mite’s interior through the cuticle, mouthparts, and anus. At 96 h, the mite’s body was fully covered with hyphae and a large number of spores, ultimately leading to the death of the host. The results of the pathogenicity tests indicated that strain HYC2101 was significantly pathogenic to both female adults and larvae, with greater pathogenicity against female adults. The LC_50_ values after 7 days of infection were 5.92 × 10⁴ and 9.22 × 10^5^ spores/mL for female adults and larvae, respectively. Under the highest spore concentration of 1 × 10^8^ spores/mL, the LT_50_ values for female adults and larvae were 2.80 and 4.79 days, respectively. In conclusion, the highly pathogenic *P. oxalicum* strain HYC2101 shows significant potential for use in the green prevention and control of citrus red mites and warrants further development as a biocontrol resource.

## 1 Introduction

*Panonychus citri*, commonly known as the citrus red mite, is a significant pest that poses severe threats to a variety of plants, including citrus (Pan et al., 2023; Basso et al., 2023), figs (*Ficus carica*) (Arabuli et al., 2016), and pears (Shao et al., 2025), with particularly pronounced damage to citrus crops. Owing to its small size, rapid reproduction rate, and strong dispersal capability, *P. citri* has emerged as a primary pest constraining the development of the citrus industry (Li et al., 2017; Gotoh et al., 2003; Vela et al., 2017). At present, chemical control remains the predominant method for managing large-scale mite infestations. However, the extensive application of chemical pesticides has triggered a host of severe issues, including excessive pesticide residues, damage to natural enemies, and disruption of ecological balance in citrus orchards, which pose environmental and social challenges. Moreover, the unique ecological strategies and environmental adaptability of mites have led to increasingly prominent problems of pesticide resistance and resurgence. Research has demonstrated that *P. citri* has developed resistance to a variety of conventional acaricides, such as spirodiclofen, cyenopyrafen, bifenazate, fenpyroximate, tolfenpyrad, etoxazole, and abamectin (Cheng et al., 2022; Pan et al., 2020; Tadatsu et al., 2022; Alavijeh et al., 2020). In light of these issues, there is an urgent need to break the deadlock caused by chemical control by developing efficient, economical, and environmentally friendly biopesticides for pest management in citrus production.

The most extensively and thoroughly studied acaricidal biocontrol fungi currently primarily originate from the class Sordariomycetes, including genera such as Beauveria, Metarhizium, and Verticillium (Folorunso et al., 2024; Islam et al., 2021). These biocontrol fungi have a relatively broad host range, covering a variety of harmful mites in the order Trombidiformes. They have been reported to be highly pathogenic to *Tetranychus urticae* (Al Khoury, 2021; Hernández-Valencia et al., 2024), *Tetranychus merganser* (Alfaro-Valle et al., 2022; Rasool et al., 2023), *Eutetranychus africanus* Tucker (Wasuwan et al., 2021), and *Tetranychus truncatus* Ehara (Chaithra et al., 2022). In addition, biocontrol fungal strains can also effectively reduce mite populations by colonizing plants (Dash et al., 2018). For example, *Beauveria bassiana* colonizing *Phaseolus vulgaris* L. can reduce the reproduction rate of *Tetranychus urticae* by 51.44% and the net reproduction rate by 42.07% (Hong et al., 2024). To date, several virulent strains against *P. citri* have been successfully isolated, including *Paecilomyces farinosus* (Long et al., 2017), *Lecanicillium lecanii* (Dyah et al., 2022), *B. bassiana*, and *Metarhizium anisopliae*. Among them, *B. bassiana* and *M. anisopliae* have achieved a mortality rate of over 85% against adult *P. citri* in laboratory settings (Qasim et al., 2021). However, it has also been found that the physiological characteristics of biocontrol fungi make them susceptible to environmental factors such as temperature and ultraviolet radiation, which limit their survival, biocontrol efficacy, and persistence (Cafarchia et al., 2022; Jaronski, 2010). Therefore, further exploration of biocontrol fungi, especially those with strong tolerance to environmental stress, will help enrich the existing acaricidal biocontrol fungal resource pool and provide more reliable support for the application of biocontrol formulations.

To effectively control *P. citri*, this study isolated a heat-resistant and highly pathogenic strain, HYC2101, from diseased *P. citri* specimens collected in Taizhou City, Zhejiang Province, China. The strain was identified based on its morphological and molecular biological characteristics. We further investigated its biological properties, infection and pathogenic mechanisms, and acaricidal activity. This research aims to provide a valuable addition to the existing biocontrol fungal resources for mites and to lay the foundation for the development of biocontrol agents against *P. citri*.

## 2 Materials and methods

### 2.1 Strain isolation and preservation

Naturally occurring *P. citri* mites were harvested from citrus orchards in Taizhou City, Zhejiang Province, China. The collected samples were placed in sterile centrifuge tubes containing 100 μL of sterile water with 0.10% Tween-80. After thorough shaking, the supernatant was serially diluted to 10^−1^, 10^−2^, and 10^−3^. These dilutions were spread onto potato dextrose agar (PDA) plates (composition per liter: 200 g potato extract, 20 g glucose and 20 g agar). The plates were inverted and incubated in the dark at 28°C in a constant-temperature incubator. Once single colonies formed, pure cultures were obtained by transferring individual colonies to fresh PDA medium. The isolated strain was preserved and designated as HYC2101 (deposited in the China General Microbiological Culture Collection Center, accession number: CGMCC 40263). Adult female *P. citri* used in this study were initially collected from citrus orchards at the Zhejiang Academy of Agricultural Sciences and have been maintained under greenhouse conditions at the institute for over 10 generations. Synchronized adult females at a uniform developmental stage were selected for inoculation experiments.

### 2.2 Colony morphology of strain HYC2101

Strain HYC2101 was inoculated onto PDA plates and incubated in the dark at 28°C for 5–14 days in a constant-temperature incubator. Colony characteristics, including pigmentation, surface texture, and other macroscopic features, were visually evaluated. For microscopic examination, the slide culture method was utilized to comprehensively document the fungal structures, including hyphal development, conidiophore morphology, and spore characteristics, using compound light microscopy (Nikon, SMZ25, Japan). Strain HYC2101 was preliminarily identified based on its morphological characteristics and on comparison with the previously reported characteristics of *P. oxalicum* (Song et al., 2024).

### 2.3 Molecular identification of strain HYC2101

The molecular identification of strain HYC2101 was conducted by sequencing the ribosomal internal transcribed spacer (ITS) region, *β*-tubulin (*BenA*), calmodulin (*CaM*), and the RNA polymerase II subunit (*RPB2*) genes. The ITS region was amplified using the primers ITS1 (5′-TCCGTAGGTGAACCTGCGG-3′) and ITS4 (5′-TCCTCCGCTTATTGATATGC-3′). For the amplification of the *BenA* gene, primers BT2a (5′-GGTAACCAAATCGGTGCTGCTTTC-3′) and BT2b (5′-ACCCTCAGTGTAGTGACCCTTGGC-3′) were used (Song et al., 2024). The CaM region was amplified with CaM-F-P (5′-GCGGAAATGAAGCCGTTG-3′) and CaM-R-P (5′-TGAGTGCCCCAAATGACGAG-3′), while the RPB2 region utilized primers RPB2-R-P (5′-GGAGACCAACAAGGAGCCAA-3′) and RPB2-F-P (5′-CGCAGTGAGTCCAGGTATGG-3′). All oligonucleotide primers were synthesized by Shanghai Bioengineering Co., Ltd. The PCR amplicons were purified and commercially sequenced by the same service provider. The resultant sequences were subjected to BLASTn homology searches against the NCBI database. Representative ITS, *BenA*, CaM and RPB2 gene sequences of related strains were downloaded from GenBank. Molecular phylogeny was reconstructed using PhyloSuite v1.2.3 software with the maximum-likelihood algorithm, and the taxonomic affiliation was confirmed through 1,000 bootstrap replications to assess nodal support values (Zhang et al., 2020; Xiang et al., 2023).

### 2.4 Biological characteristics of strain HYC2101

#### 2.4.1 Influence of various culture media on mycelial growth and sporulation capacity of strain HYC2101

Following 7 days of streak-plate cultivation on agar medium, spores of strain HYC2101 were harvested via the addition of sterile distilled water and delicate scraping of the colony surface. The collected spore suspension was subsequently filtered through sterile gauze to eliminate hyphal fragments and adjusted to a concentration of 1 × 10^5^ spores/mL. For the preparation of inoculated plates, 1,000 μL of the spore suspension was aseptically incorporated into liquefied PDA medium at approximately 45°C prior to solidification. Utilizing a sterile cork borer, 0.50 cm mycelial plugs were aseptically transferred onto solid PDA, PSA (potato sucrose agar medium: boiling supernatant of 200 g potato, 20 g sucrose, 20 g agar per liter), SDAY (sabouraud dextrose yeast extract agar medium: 10 g yeast extract, 40 g dextrose, 10 g peptone, 20 g agar per liter, pH 6.0), MEA (malt extract agar medium: 30 g malt extract, 3 g soy peptone, 15 g agar per liter, pH 5.6), CDA (czapek-dox agar medium: 3 g NaNO_3_, 1.0 g KH_2_PO_4_, 0.5 g MgSO_4_ · 7H_2_O, 0.5 g KCl, 0.01 g FeSO_4_, 30 g sucrose, 15 g agar per liter, pH 7.3), and MCDA (modified Czapek-Dox agar medium: 1 g glucose, 0.2 g MgSO₄ · 7H₂O, 0.2 g KCl, 1 g KH₂PO₄, 2 g NaNO₃, 0.2 g yeast extract, 15 g agar per liter, pH 6.6–7.0) media. Cultures were incubated at 28°C in complete darkness, with three replicates for each treatment. Daily observations were conducted over a 10-day period, during which colony diameters were measured employing the perpendicular cross method. Sterile mycelial plugs (0.50 cm in diameter) were aseptically inoculated onto both solid and liquid media formulations. The corresponding liquid media (designated as PDAL, PSAL, SDAYL, MEAL, CDAL, and MCDAL) were prepared by omitting agar from their respective solid formulations. Solid media plates were incubated at 28°C in complete darkness for 10 days. Post-incubation, spores were eluted by adding 10 mL of sterile distilled water to each plate and gently scraping the colony surface. The resultant suspension was filtered through sterile gauze to remove hyphal debris. Liquid cultures were cultivated in a rotary shaker at 28°C with agitation at 160 rpm for 10 days. Mycelia were subsequently removed by filtration through sterile Miracloth, after which the spore suspension was collected for quantification. Each treatment was performed in triplicate. Spore counts were determined using an improved Neubauer hemocytometer.

#### 2.4.2 Influence of incubation duration on spore germination of strain HYC2101

Spores were harvested from a 7-day streak-cultured plate of strain HYC2101 by washing with PDA liquid medium, and the mycelia were filtered out to obtain the spore suspension. After adjusting the concentration to 1 × 10^5^ spores/mL, 5.00 μL of the suspension was mounted onto a sterile microscope slide. Each slide was positioned on a U-shaped glass tube within a sterile petri dish, with sterile moist filter paper at the bottom to maintain humidity. The setup was incubated at 28°C in the dark. Samples were collected at 3, 4, 5, 6, and 7 h post-inoculation for microscopic observation of spore germination. At each time point, at least 100 spores were examined to calculate the germination rate based on the formula: Germination rate (%) = (Number of germinated spores/Total number of spores) × 100. Each treatment was performed with three biological replicates.

#### 2.4.3 Impact of temperature on mycelial growth and spore germination of strain HYC2101

Mycelial plugs (0.50 cm in diameter) were aseptically cut using a cork borer and placed at the center of PDA plates. The inoculated plates were inverted and incubated in the dark at temperatures of 10, 15, 20, 25, 30, 35, and 40°C, with three replicates per temperature. Mycelial growth rate was measured following the method in Section 2.4.1. Spore suspensions were incubated in the dark at constant temperatures of 20, 25, 30, 35, 40, and 45°C. After 6 h, samples were taken to assess spore germination, with three replicates per temperature. Spore suspension preparation and germination rate determination followed the procedures in Section 2.4.2.

### 2.5 Assessment of biocontrol efficacy of strain HYC2101

This assessment adapted the methodology from Zhang et al. (2023). Citrus leaves with newly emerged adult female *P. citri* were selected and immersed in a spore suspension of strain HYC2101 (1.0 × 10^8^ spores/mL with 0.1% Tween-80) for 5 s. After air-drying, approximately 40 adult female mites were transferred to surface-sterilized citrus leaves, with Vaseline applied to the leaf edges to prevent mite escape. The leaves were placed in Petri dishes lined with sterile moist filter paper. The dishes were incubated in a controlled-environment growth chamber set at 30 ± 1°C, 14 h light/10 h darkness, and 75 ± 5% relative humidity. Samples were collected at 12, 24, 48, 72, and 96 h post-inoculation to examine *P. citri* cuticular infection using a stereomicroscope (Nikon DS-Ri2, Japan). *P. citri* specimens were fixed in 2.5% glutaraldehyde, washed with PBS, post-fixed with 1% osmic acid, dehydrated with graded ethanol series, treated with isoamyl acetate, and critical point-dried. After sputter-coating with gold, infection patterns on the mite cuticle were characterized using scanning electron microscopy (SEM, Hitachi, SU8100, Japan).

### 2.6 Laboratory bioassays of strain HYC2101 against *P. citri*

Bioassays against *P. citri* were performed according to Section 2.5. Synchronized mites (larvae, nymphs, and 1- to 2-day old adults) were inoculated onto citrus leaf discs (35–45 mites/disc). Eggs were collected by allowing adults to oviposit for 24 h, yielding 24–30 eggs/disc after adult removal. Six treatments per developmental stage included: five conidial suspension concentrations (10⁴–10^8^ spores/mL in 0.1% Tween-80) and a negative control (sterile water with 0.1% Tween-80), each with three biological replicates. Mortality of motile stages was recorded daily from days 2 to 8 post-inoculation, with infection confirmed by mycosis development. For eggs, daily hatch counts were recorded until no emergence occurred for two consecutive days. Cumulative hatchability (%) = (Cumulative number of hatched eggs/Total eggs tested) × 100.

### 2.7 Statistical analyses of experimental data

All data were normalized before analysis. Levene’s test was used to assess variance homogeneity, and Tukey’s honestly significant difference (HSD) test was used to determine significant differences. Results are expressed as mean ± standard deviation, with a significance level of *α* = 0.05. LT_50_ and LC_50_ values along with their 95% confidence intervals (CIs) were calculated using probit analysis. All statistical analyses were performed using SPSS 22.0 (International Business Machines Corporation) and GraphPad Prism 8.0 (GraphPad Software, Inc.).

## 3 Results

### 3.1 Identification and characterization of the isolate

**Figure 1:**
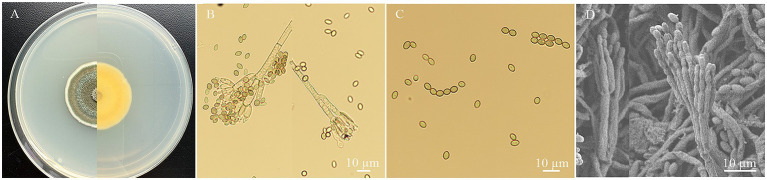
The morphology characteristics of *P. oxalicum* HYC2101 isolate on PDA. **(A)** Upper and dorsal surfaces of HYC2101 strain colony on PDA at day 7. **(B)** Sporophore with developing conidia under a light microscope (×400); **(C)** Conidia and chains of conidia under a light microscope (×400); **(D)** Sporophore with conidia under scanning electron microscopy (×500).

Strain HYC2101 exhibited the following cultural characteristics on PDA medium: It grew relatively slowly, developing to a diameter of about 3.04 cm after 7 days of cultivation. The colony was circular, flat, and velvety in texture. Mycelium initially appeared white, later turning dark green with abundant spore production. The colony’s reverse side showed pale yellow pigmentation. Under an optical microscope, strain HYC2101 displayed the morphological features shown in Figure 1A. Conidiophores bore biverticillate (two-tiered) penicilli, sometimes monoverticillate (single-tiered) or terverticillate (three-tiered). Each whorl comprised 1–3 metulae, sized 3.30⁓4.12 × 16.96⁓22.86 μm. Phialides were slender and flask-shaped, with 4–8 per whorl, measuring 2.08⁓3.41 × 8.38⁓12.40 μm, and bore conidial chains at their tips (Figures 1B,D). Conidia were elliptical to subglobose, greenish-blue, and sized 2.12⁓3.43 × 3.69⁓4.94 μm. Conidial chains were cylindrical (Figures 1C,D).

**Figure 2:**
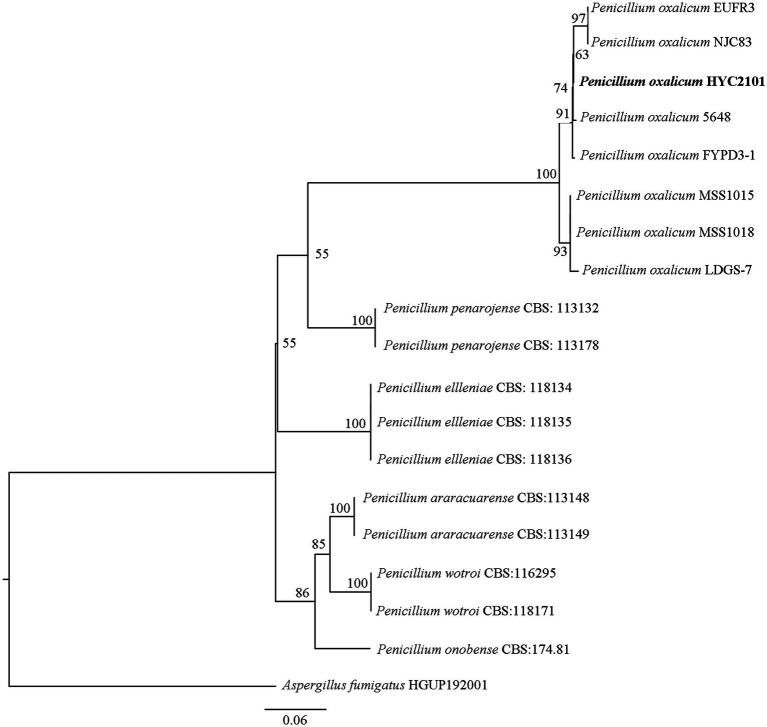
Phylogenetic tree based on concatenated ITS, *BenA, CaM* and *RPB2* sequences of the strain HYC2101. The phylogenetic tree based on concatenated ITS, *BenA*, *CaM* and *RPB2* sequences of strain HYC2101 was reconstructed using Maximum-Likelihood (ML) analysis in PhyloSuite v1.2.3, with *Aspergillus fumigatus* (HGUP192001) as the outgroup. The clade supports were performed with the standard nonparametric bootstrap analysis of 1,000 replicates.

DNA sequencing of the ITS, *BenA*, *CaM* and *RPB2* genes of strain HYC2101 generated fragments of 572 bp, 457 bp, 581 bp and 916 bp, respectively. BLAST comparison of these sequences in GenBank revealed that strain HYC2101 was 100% homologous to *P. oxalicum*. The ITS, *BenA*, *CaM* and *RPB2* sequences were concatenated to construct a Maximum-Likelihood phylogenetic tree. The resulting phylogenetic analysis indicated that strain HYC2101 clustered stably within the *P. oxalicum* clade (Figure 2), thereby corroborating our morphological identification of the HYC2101 isolate as *P. oxalicum*. Detailed information on the strains used in the phylogenetic analysis is provided in Supplementary Table S1.

### 3.2 Influence of culture media on mycelial growth and sporulation in strain HYC2101

**Figure 3:**
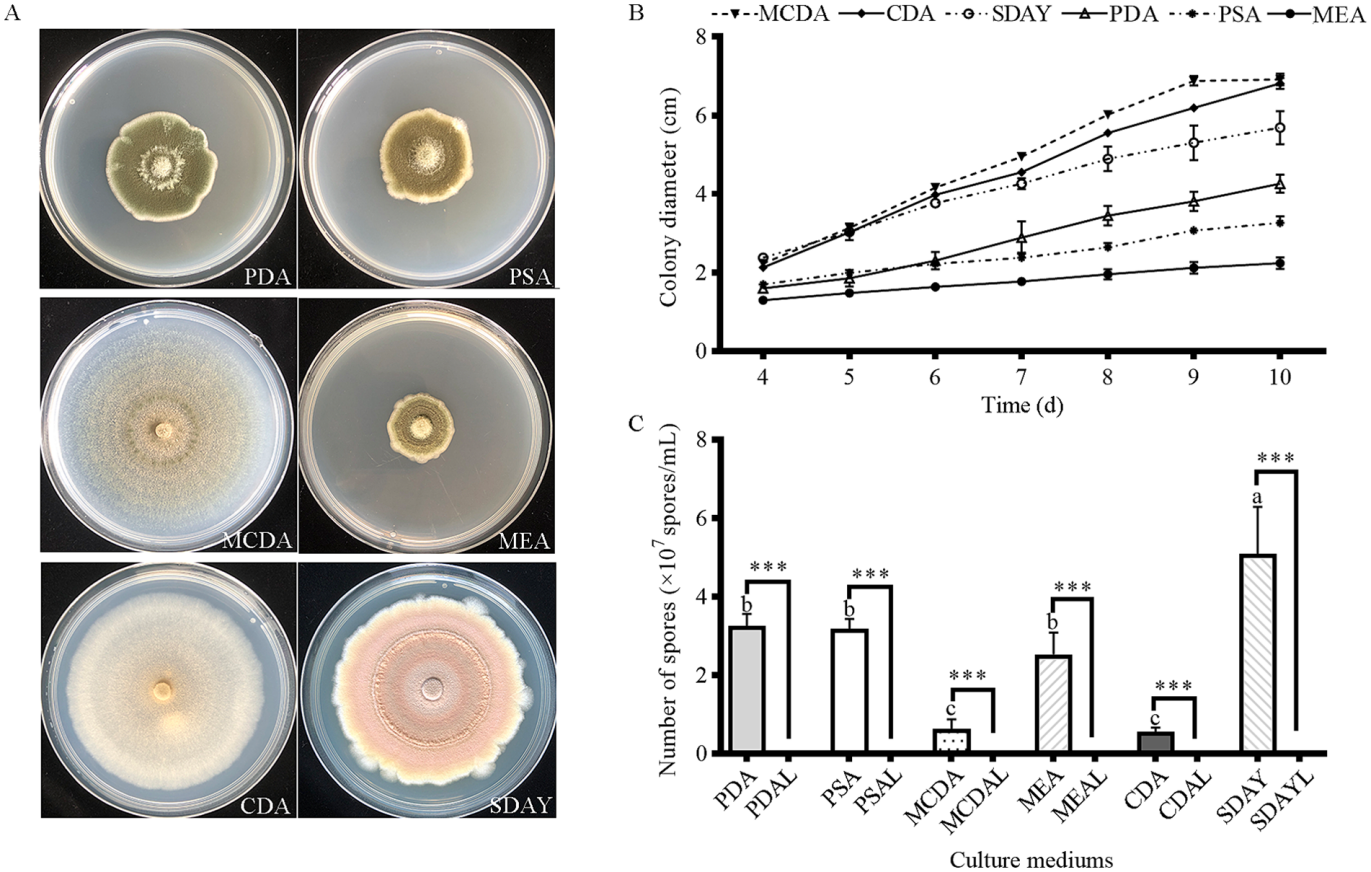
Mycelial growth and sporulation yield of strain HYC2101 on different culture media. **(A)** Growth phenotypes of strain HYC2101 on various media (9 d); **(B)** Effects of different media on mycelial growth of HYC2101; **(C)** Effects of different media on sporulation yield of HYC2101. All data are presented as mean ± standard deviation (*n = 3). Lowercase letters indicate multiple comparisons of sporulation yields among solid media, while *** denote statistically significant differences (*p* < 0.001, Student’s *t*-test) between solid and liquid media.

The colonial morphology of strain HYC2101 varied significantly across different culture media. On SDAY medium, colonies exhibited an orange-red hue, whereas on CDA, they developed a white appearance. When grown on PSA, MEA, and MCDA media, the colonies displayed pigmentation ranging from grass-green to olive-green (Figure 3A). Strain HYC2101 demonstrated robust growth on all tested media (PSA, SDAY, MCDA, MEA, and CDA), with the highest growth rate observed on MCDA medium. After 9 days of cultivation, the colony diameter on MCDA medium reached 6.92 cm (Figure 3B). In terms of sporulation, SDAY medium produced the highest spore yield (5.08 × 10^5^ spores/mL), significantly exceeding that of other solid media. Notably, minimal sporulation was observed in any of the six liquid media tested (Figure 3C).

### 3.3 Influence of incubation time on spore germination of strain HYC2101

**Figure 4:**
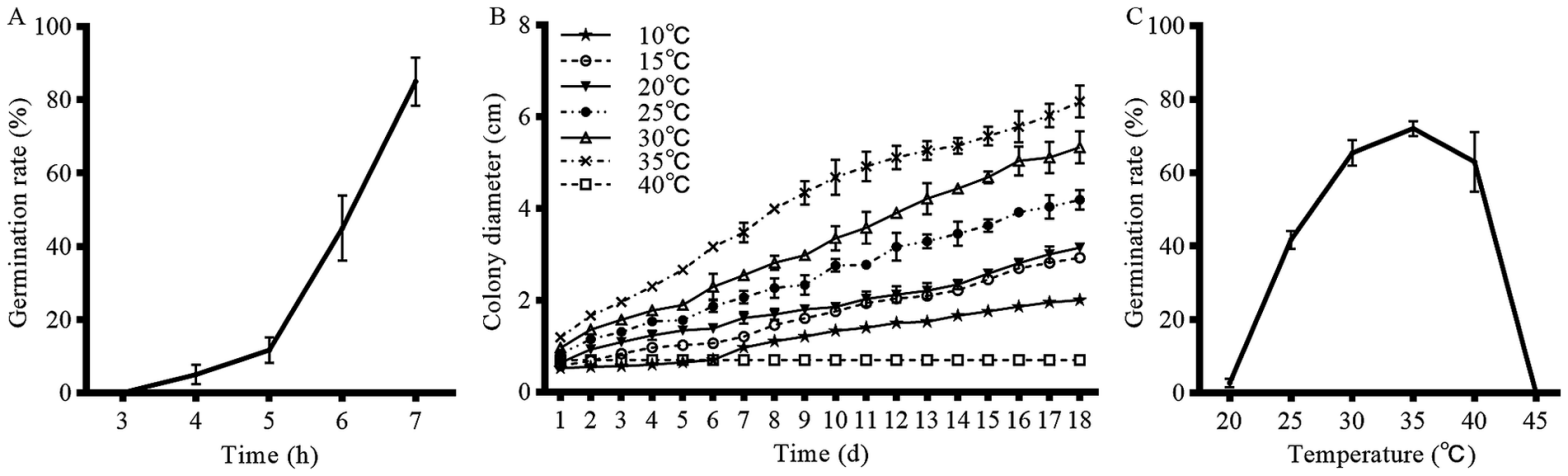
Effects of temperature and incubation time on mycelial growth and conidial germination of strain HYC2101. **(A)** Effect of time on conidial germination rate of strain HYC2101. **(B)** Effect of temperature on mycelial growth of strain HYC2101. **(C)** Effect of temperature on conidial germination rate of strain HYC2101.

Spores of strain HYC2101 were incubated at 28°C, and the germination rate was assessed at 3, 4, 5, 6, and 7 h post-inoculation. As depicted in Figure 4A, the germination rate of strain HYC2101 spores increased progressively with extended incubation time. Notably, after 7 h of incubation, the germination rate exceeded 85%, indicating robust germination activity.

### 3.4 Thermal adaptation and germination efficiency of strain HYC2101

Strain HYC2101 demonstrated a broad temperature tolerance range on PDA medium, with mycelial growth observed between 10⁓35°C. Notably, the mycelial growth rate was significantly elevated at temperatures between 25 and 35°C, compared to other tested ranges (Figure 4B). Temperature had a substantial impact on the spore germination rate of strain HYC2101. After 6 h of incubation, the optimal temperature range for spore germination was identified as 25⁓40°C, with 35°C being the most favorable (Figure 4C). Minimal germination was observed below 20°C or above 45°C. Collectively, the optimal temperature range for both mycelial growth and spore germination of strain HYC2101 was 25⁓35°C, with peak performance at 35°C, highlighting its notable heat tolerance.

### 3.5 Histopathological analysis of *P. citri* infected with strain HYC2101 spores

**Figure 5:**
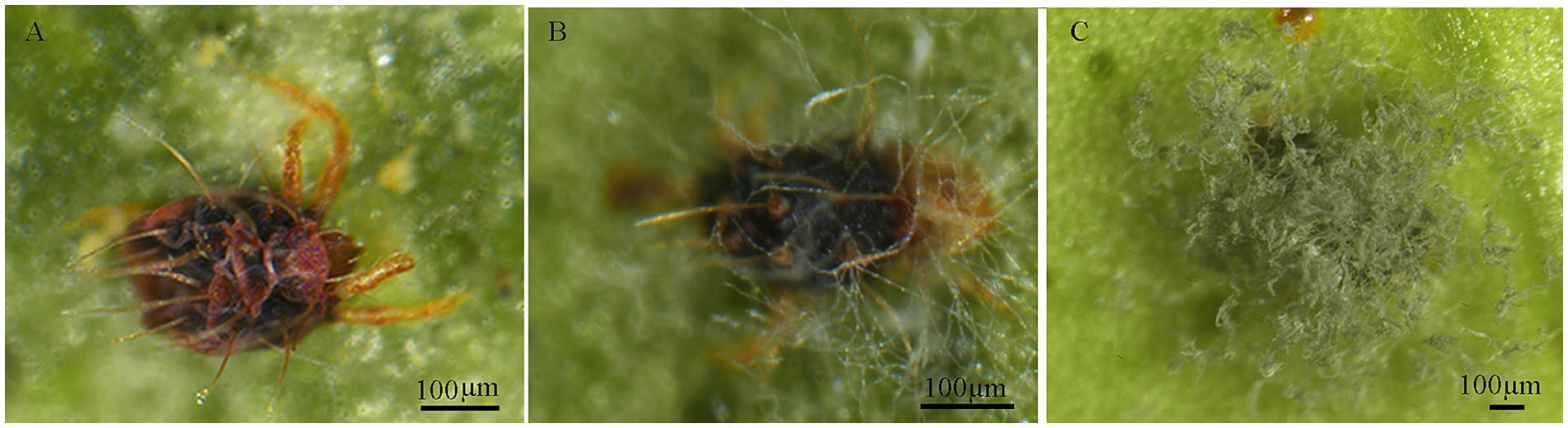
External symptoms on *P. citri* infected with *P. oxalicum* HCY2101. **(A)** *P. citri* at 48 h post-infection. **(B)** *P. citri* at 72 h post-infection. **(C)** *P. citri* at 96 h post-infection.

Stereomicroscopic observations revealed that 48 h post-inoculation of *P. citri* adult females with strain HYC2101 conidial suspensions, the mites exhibited sluggish movement, with sparse mycelial growth emerging on their dorsal surfaces and legs (Figure 5A). After 72 h, extensive white mycelial growth was observed across the entire body of the adult mites (Figure 5B). By 96 h, the mites were completely enveloped by fungal hyphae, with abundant sporulating structures and green conidia forming on the mycelial mats (Figure 5C).

**Figure 6:**
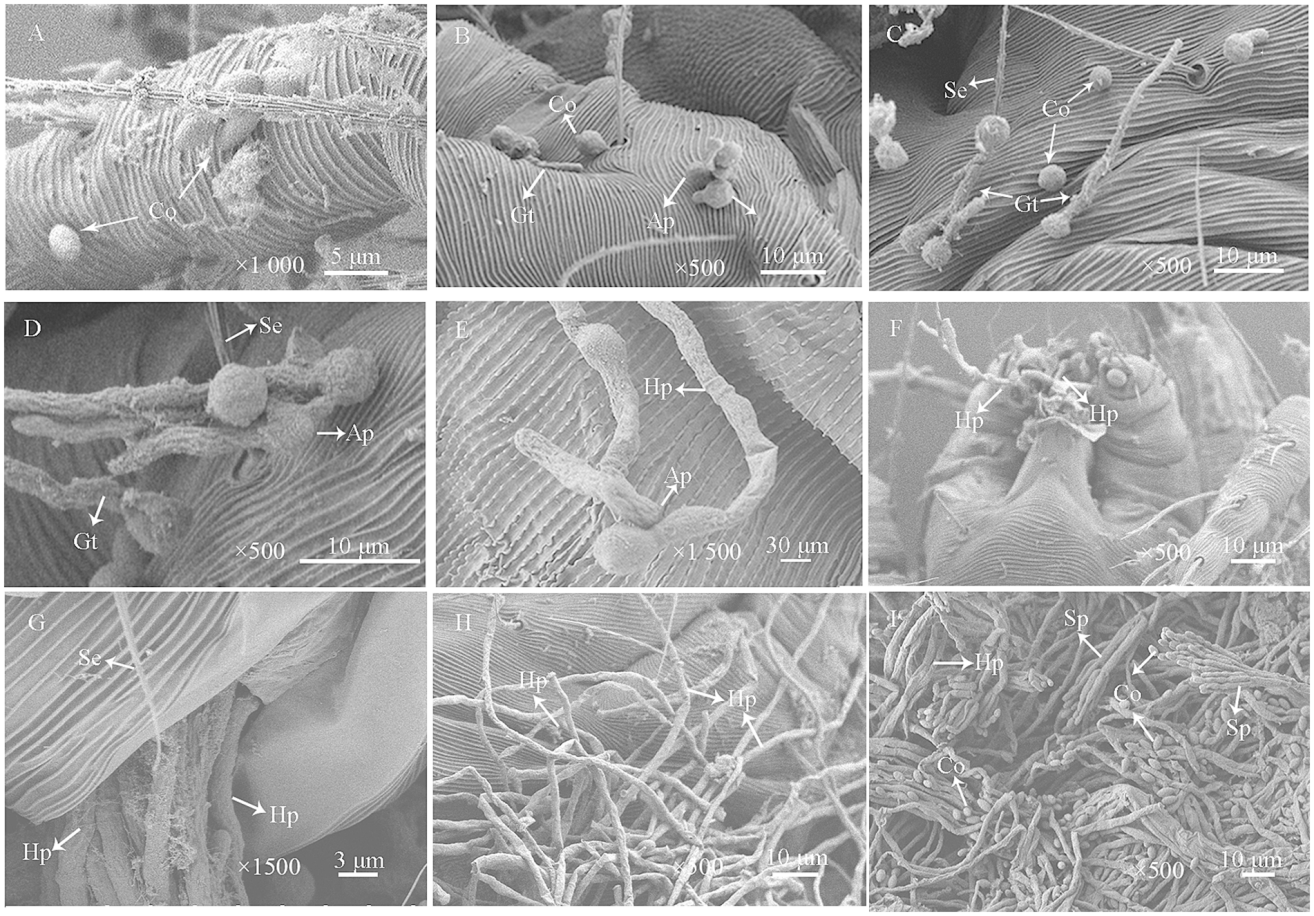
Scanning electron micrographs of *P. citri* adults infected with *P. oxalicum* HCY2101. **(A)** Conidial (Co) adhesion to the mite’s cuticle and setae (Se) during early infection. **(B,C)** Some conidia germinated and produced germ tubes (Gt) (24 h). **(D)** Germ tubes grew along the cuticle and formed appressoria (Ap) for penetration (24 h). **(E)** Hyphae (Hy) extended across the cuticle with appressoria breaching the epidermis (48 h). **(F)** Hyphal invasion through the mouthparts of *P. citri* (48 h). **(G)** Hyphal penetration via the anus (48 h). **(H)** Mycelial coverage on the mite’s cuticle (72 h). **(I)** Extensive hyphal colonization and sporulation on the cuticle (96 h).

Under SEM, during the initial inoculation stage, conidia adhered to the mite cuticle, particularly in surface folds, grooves, and setal bases (Figure 6A). Within 24 h post-inoculation, the conidia germinated, producing germ tubes and appressoria (Figures 6B–D). Some germ tubes directly penetrated the cuticle through enzymatic degradation (Figures 6B,D). After 48 h, the germ tubes further developed into hyphae, exhibiting distinct invasion patterns: some extended along the cuticle, others penetrated the body wall directly, and a subset entered through natural openings such as the hypostome and anus (Figures 6E–G). After 72 h, dense mycelial networks extensively covered the mite’s surface (Figure 6H). By 96 h, the mite cadavers were completely overgrown by fungal hyphae, which further developed into aerial mycelia bearing abundant sporulating structures and conidia (Figure 6I).

### 3.6 Pathogenicity to *P. citri* eggs

**Figure 7:**
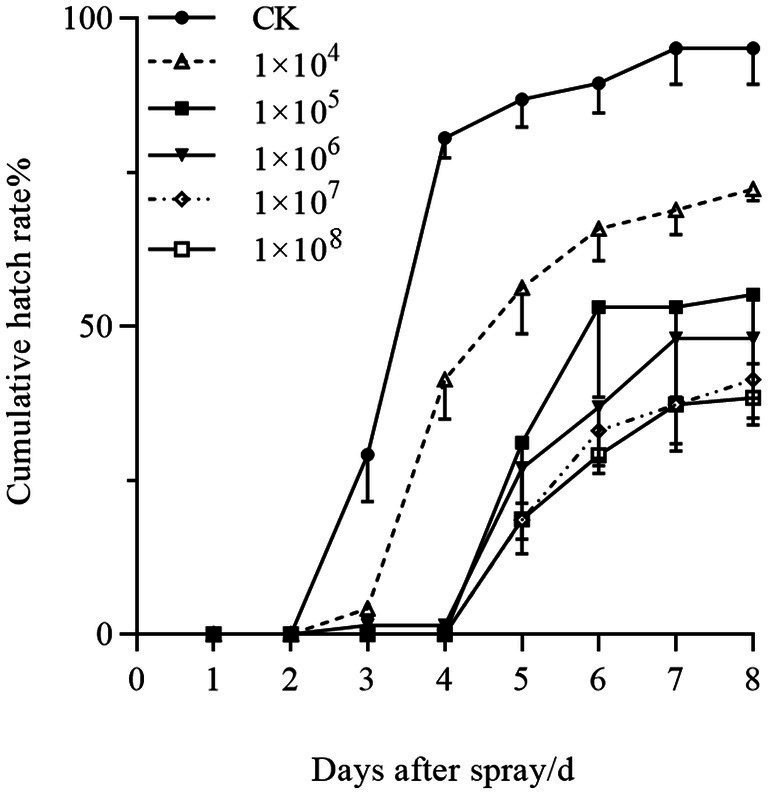
Concentration-dependent effects of *P. oxalicum* HYC2101 spores on cumulative egg hatchability in *P. citri*.

Pathogenicity assessment of strain HYC2101 against *Panonychus citri* eggs revealed significant delays in hatching initiation and suppression of final hatchability (Figure 7). Control eggs commenced hatching on day 3, while at concentrations >1 × 10^5^ spores/mL, treated eggs exhibited postponed hatching initiation until day 5. Furthermore, although cumulative hatchability in treated groups increased temporally, it remained significantly lower than controls, with higher concentrations inducing greater inhibition. At day 8 post-treatment, hatchability was 38.38% at 1 × 10^8^ spores/mL (see Figure 7).

### 3.7 Pathogenicity against adults, nymphs and larvae stages of *P. citri*

Bioassays conducted with strain HYC2101 demonstrated that mortality in *P. citri* adults, nymphs and larvae was dependent on both time and spore concentration. The lowest LC_50_ values were observed at 7 days post-inoculation, with adults showing an LC_50_ of 5.92 × 10⁴ spores/mL, nymphs showing an LC_50_ of 1.79 × 10^6^ spores/mL, and larvae showing an LC_50_ of 9.22 × 10^5^ spores/mL (Table 1). A negative correlation was noted between spore concentration and LT_50_ values. Specifically, at spore concentrations ranging from 1 × 10^8^ to 1 × 10^5^ spores/mL, adult females exhibited significantly faster mortality (LT_50_: 2.80–6.40 days) compared to nymphs (LT_50_: 5.12–7.73 days) and larvae (LT_50_: 4.79–7.28 days) (Table 2). These findings demonstrate that strain HYC2101 exhibits significantly higher pathogenicity against adult females of *P. citri* than against nymphs and larvae. The reduced mortality in immature stages may be linked to their abbreviated developmental windows and cuticular restructuring during molting. Owing to its high pathogenicity against *P. citri*, the strain emerges as a prime candidate for field evaluation in citrus mite biocontrol programs.

**Table 1:** Pathogenicity regression equations for LC_50_ values of *P. oxalicum* HCY2101 against *P. citri.*

Developmental stage	Days	LC_50_ (spores/mL)	Regression equation (*y*=)	95%CI (spores/mL)	*p*
Adult	3	1.13 × 10^8^	*y* = 0.283*x*−2.277	4.31 × 10^7^–4.43 × 10^8^	0.587
	4	2.94 × 10^6^	*y* = 0.352*x*−2.277	1.74 × 10^6^–5.23 × 10^6^	0.671
	5	7.41 × 10^5^	*y* = 0.321*x*−1.867	6.66 × 10^5^–3.66 × 10^6^	0.882
	6	1.67 × 10^5^	*y* = 0.365*x*−1.906	9.00 × 10^4^–2.86 × 10^5^	0.232
	7	5.92 × 10^4^	*y* = 0.356*x*−1.699	2.17 × 10^4^–1.27 × 10^5^	0.081
Nymphs	3	8.50 × 10^10^	*y* = 0.294*x*−3.215	5.25 × 10^9^–2.40 × 10^13^	0.535
	4	1.42 × 10^9^	*y* = 0.276*x*−2.523	2.70 × 10^8^–2.35 × 10^10^	0.331
	5	2.10 × 10^8^	*y* = 0.239*x*−1.986	5.05 × 10^7^–2.30 × 10^9^	0.597
	6	7.90 × 10^6^	*y* = 0.219*x*−1.511	2.86 × 10^6^–3.35 × 10^7^	0.291
	7	1.79 × 10^6^	*y* = 0.227*x*−1.421	7.16 × 10^5^–5.16 × 10^6^	0.463
Larvae	3	5.92 × 10^10^	*y* = 0.226*x*−2.438	4.2 × 10^9^–8.99 × 10^12^	0.570
	4	1.87 × 10^9^	*y* = 0.248*x*−2.297	3.58 × 10^8^–2.88 × 10^10^	0.988
	5	3.47 × 10^7^	*y* = 0.237*x*−1.783	1.72 × 10^7^–1.49 × 10^8^	0.695
	6	2.98 × 10^6^	*y* = 0.232*x*−1.504	1.32 × 10^6^–7.76 × 10^6^	0.290
	7	9.22 × 10^5^	*y* = 0.229*x*−1.366	3.22 × 10^5^–2.62 × 10^6^	0.137

*p* > 0.05 showed good regression fit to the probit model.

**Table 2:** Pathogenicity regression equations for LT_50_ values of *P. oxalicum* HCY2101 against *P. citri.*

Developmental stage	Conidia concentration (spores/mL)	LT_50_ (d)	Regression equation (*y*=)	95%CI (d)	*p*
Adult	10^8^	2.80	*y* = 3.379*x*−1.512	2.54–3.03	0.053
	10^7^	3.91	*y* = 2.968*x*−1.756	3.68–4.13	0.811
	10^6^	4.78	*y* = 2.766*x*−1.878	4.49–5.11	0.905
	10^5^	6.40	*y* = 2.609*x*−2.104	5.92–7.07	0.586
Nymphs	10^8^	5.12	*y* = 3.724*x*−2.649	4.81–5.48	0.284
	10^7^	6.52	*y* = 3.101*x*−2.525	6.01–7.31	0.508
	10^6^	6.77	*y* = 3.702*x*−3.074	6.18–7.74	0.148
	10^5^	7.73	*y* = 4.268*x*−3.790	7.12–8.70	0.673
Larvae	10^8^	4.79	*y* = 2.955*x*−2.011	4.47–5.21	0.273
	10^7^	5.92	*y* = 2.817*x*−2.175	5.50–6.47	0.989
	10^6^	6.47	*y* = 3.125*x*−2.535	6.00–7.11	0.745
	10^5^	7.28	*y* = 3.615*x*−3.117	6.77–7.80	0.150

## 4 Discussion

In this study, strain HYC2101 was successfully isolated from diseased specimens of *P. citri*. Through comprehensive multilocus phylogenetic analyses (ITS, BenA, CaM, and RPB2) alongside cultural and morphological assessments (Song et al., 2024), strain HYC2101 was definitively classified as *P. oxalicum*. Sporulation characteristic investigations revealed that strain HYC2101 exhibited optimal conidial production on SDAY solid medium, with a notable reduction in sporulation efficiency in liquid culture environments. Comparable results for *P. oxalicum* BGPUP-4 Singh et al. (2018) demonstrated that solid-state fermentation significantly enhanced conidial yield of strain HYC2101 compared to liquid fermentation processes. Collectively, these data established solid-state fermentation as the superior method for large-scale industrial cultivation of strain HYC2101. Pathogenicity assays validated that strain HYC2101 demonstrated substantial virulence against adult female *P. citri*, achieving an LC_50_ of 5.92 × 10^4^ spores/mL 7 days post-inoculation. The conclusive identification of strain HYC2101 as *P. oxalicum*, along with its superior conidial production via solid-state fermentation and marked pathogenicity toward *P. citri*, suggested its potential as a promising biological control strain for *P. citri*.

Studies on biological characteristics indicate that strain HYC2101 achieves maximal mycelial growth and spore germination within the temperature range of 25–35°C, with optimal performance at 35°C. This thermal adaptation markedly differs from that of commonly utilized biocontrol fungi such as *Metarhizium* spp., *Beauveria* spp., and *Isaria* spp., which typically exhibit optimal growth at 20–28°C. The growth of these fungi becomes restricted above 30°C and shows significant inhibition beyond 34°C (Seib et al., 2023; Reyes-Haro et al., 2024; Li et al., 2024). Thus, strain HYC2101 demonstrates exceptional thermotolerance. Given that viable propagules (conidia/mycelia) represent the fundamental active components in biocontrol formulations, this thermal stability is likely to enhance formulation stability, environmental adaptability, and field persistence of the resulting products (Tong and Feng, 2020; Kryukov et al., 2018; Gasmi et al., 2021; Arévalo Rojas et al., 2023). Currently, the predominant method for acquiring thermotolerant biocontrol strains involves screening wild isolates with heat-resistant phenotypes from natural environments. Paixão et al. (2023) reported that the tick-pathogenic strain *Metarhizium anisopliae* CG47 exhibited significant thermal tolerance at 32°C. In another study (Mseddi et al., 2022), *Beauveria bassiana* B12 conidia retained 72% germination viability after 2 h of exposure to 45°C. During the screening of biocontrol agents against the rice stem borer (*Chilo suppressalis*), only 4 of 15 entomopathogenic fungal strains (e.g., *B. bassiana* BBLN1 and *Metarhizium anisopliae*. MASA) demonstrated moderate thermotolerance (conidial germination rate: 30–60%) following 2 h at 45°C (Shahriari et al., 2021). Beyond natural screening, optimizing fermentation processes may enhance the thermal resilience of biocontrol strains (Lima et al., 2024; Tong and Feng, 2020; Dias et al., 2021). In recent years, multi-omics technologies have provided novel insights into the mechanisms of thermotolerance and have enabled targeted genetic modification of fungal strains. Research has revealed that copy number variations (CNVs) in photolyase and cyclophilin B genes are significantly associated with virulence and thermotolerance in *B. bassiana* (Gasmi et al., 2021). The highly thermotolerant strain *Trichoderma longibrachiatum* TaDOR673 mitigates heat stress through MAPK signaling and heat-shock response pathways (Poosapati et al., 2021). Given the superior thermotolerance of strain HYC2101, subsequent research will focus on elucidating its stability and pathogenicity under high-temperature conditions, developing high-efficacy biocontrol formulations, and systematically evaluating the thermal resilience of these formulations as well as their sustained efficacy against field populations of *P. citri* under heat stress.

This study examined the pathogenic process of *P. oxalicum* strain HYC2101 infecting adult female *P. citri*. During the initial infection phase, the majority of conidia specifically adhered to the setae on the legs and cuticular folds of the mites. Concurrently, the oral cavity and anal opening served as the primary entry points for fungal invasion into the host. Effective conidial attachment to the mite cuticle is a critical first step for successful infection. However, the spatial distribution of these attachment sites significantly differs from the mechanisms reported for other entomopathogenic fungi infecting various insect hosts (Zhang et al., 2018; Xiang et al., 2024). When infecting aphids, *P. oxalicum* conidia predominantly adhere to the abdominal tail bristles, scaly hairs on the head and abdomen, and legs (Song et al., 2024). During infection of *Brevipalpus phoenicis* by *Hirsutella thompsonii* ESALQ-1269, conidia mainly attach to the integument, lateral propodosomal depressions, and hysterosoma (Rossi-Zalaf and Alves, 2006). For *Isaria cateniannulata* infecting *Tetranychus urticae*, conidial adhesion is concentrated on the abdomen and legs (Zhang et al., 2018). These differences indicate strain–host specificity in conidial adhesion patterns. SEM observations further revealed that germinating conidia formed appressoria and penetration pegs on the host cuticle during the early infection stage. Supporting hyphae induced localized cuticular deformation and tissue dissolution, accompanied by the deposition of a viscous extracellular matrix. This coordinated physico-enzymatic invasion strategy, mediated by focused mechanical force and the secretion of cuticle-degrading enzymes (e.g., chitinases, proteases, and hydrolytic enzymes), is a key evolutionary adaptation of entomopathogenic fungi for efficient host penetration. Our findings align with this established model (Zhang et al., 2022; Zhang et al., 2018). Notably, target pests can activate defense mechanisms when subjected to biocontrol fungal infection (Ma et al., 2024; Wang et al., 2021). For example, mosquitoes infected by *B. bassiana* use microRNAs (miRNAs) to induce cross-kingdom silencing of pathogenicity-related genes, thereby enhancing infection resistance (Wang et al., 2021). Given the demonstrated potential of strain HYC2101 as a biocontrol agent against *P. citri*, ensuring its field stability and long-term efficacy requires ongoing research into the resistance evolution dynamics of *P. citri* to this strain. Such research will provide the essential foundation for developing science-based resistance management strategies.

*Penicillium oxalicum* holds promise for the biological control of various agricultural pests. Research has shown that a spore suspension of *P. oxalicum* QLhf-1 (1 × 10^7^ spores/mL) achieved an aphid control efficacy of 89.5% after 5 days of treatment, while its fermentation broth also reached a control efficacy of 86.20% (Song et al., 2024). Additionally, the application of *P. oxalicum* 201,888 to the roots of potato varieties “Mona Lisa” and “Desiree” significantly reduced the hatching rate of *Globodera pallida juveniles* by 98.6 and 74.1%, respectively (Martinez-Beringola et al., 2013). In this study, *P. oxalicum* HYC2101 demonstrated lethal effects on both adult females and larvae of *P. citri*, with stronger pathogenicity against adult females. The LC_50_ value for adult females after 7 days of infection was 5.92 × 10⁴ spores/mL, while larvae exhibited lower sensitivity with an LC_50_ value of 9.22 × 10^5^ spores/mL. Compared to other biocontrol strains, *Paecilomyces farinosus* nq1-1 had an LC_50_ value of 7.51 × 10^8^ spores/mL for adult female *P. citri* on day 7 (Long et al., 2017), showing significantly lower pathogenicity than strain HYC2101. Furthermore, after 9 days of treatment with *Beauveria bassiana* BFZ0409 and D1344, the LC_50_ for adult female *P. citri* was 1 × 10⁴ spores/mL (Qasim et al., 2021). *Isaria cateniannulata* HL3, after 7 days of inoculation, had an LC_50_ of 3.4 × 10^4^ spores/mL for adult female *P. citri*, with pathogenicity comparable to strain HYC2101 and similarly higher pathogenicity against adult females than larvae (Li, 2023). In summary, strain HYC2101 shows superior or comparable pathogenicity to reference biocontrol strains and has acaricidal activity against all developmental stages of *P. citri*, indicating great potential for field application. Importantly, the successful development and use of fungal biocontrol agents require a systematic evaluation of their non-target effects, especially on beneficial insects and natural enemies (Cappa et al., 2022). Although this strain was isolated from citrus orchards and is widespread in natural ecosystems, its overall impact on orchard ecological communities is still not fully understood. Future studies should look into the effects of HYC2101 on beneficial arthropod communities in citrus agroecosystems. These communities include pollinators (e.g., *Apis mellifera*), predatory mites (e.g., *Amblyseius cucumeris*), and parasitoid wasps (e.g., *Encarsia formosa*). This research offers guidance for the ecologically safe use of HYC2101.

## 5 Conclusion

This study isolated *P. oxalicum* strain HYC2101, demonstrating broad temperature adaptation, strong thermotolerance, high sporulation capacity, and easy cultivability, with infection assays confirming pathogenicity against all *P. citri* developmental stages—particularly female adults. Given these attributes combined with biocontrol advantages (environmental safety, low resistance risk, economic sustainability) and China’s lack of commercial *P. oxalicum* formulations, HYC2101 exhibits significant industrialization potential; subsequent research prioritizes solid-state fermentation optimization, microbial powder formulation development, and non-target ecological assessment (bees/soil microbiota) to advance IPM integration against citrus red mites.

## Data Availability

The datasets presented in this study can be found in online repositories. The names of the repository/repositories and accession number(s) can be found in the article/Supplementary material.
