# Supplementary material for: Identification of a Penicillium oxalicum fungus isolate and its pathogenicity against Panonychus citri (McGregor)
Source: Front Microbiol. 2025 Aug 6;16:1619976. doi: 10.3389/fmicb.2025.1619976 (PMC12364956; doi:10.3389/fmicb.2025.1619976)
Supplement: Supplementary file 1 [file Table_1.docx]

| Strain | ITS | Beta | Cam | RPB2 |
| --- | --- | --- | --- | --- |
| *Penicillium oxalicum* HYC2101 | PV474179.1 | PV605618.1 |  |  |
| *Penicillium oxalicum* FYPD3-1 | PQ269782.1 | PQ516234.1 | PQ516219.1 | PQ516267.1 |
| *Penicillium oxalicum* isolate LDGS-7 | PQ606601.1 | PQ611758.1 |  | PQ611759.1 |
| *Penicillium oxalicum* strain MSS1015 | MZ157141.1 |  | MZ220653.1 | MZ190932.1 |
| *Penicillium oxalicum* strain MSS1018 |  | MZ220694.1 | MZ220657.1 | MZ190922.1 |
| *Penicillium oxalicum* strain 5648 | KJ527449.1 | KJ527414.1 |  | KJ527379.1 |
| *Penicillium oxalicum* strain NJC83 | OR054155.1 | OR249978.1 | OR255949.1 |  |
| *Penicillium oxalicum* isolate EUFR3 | KX865276.1 | KX865280.1 |  |  |
| *Penicillium penarojense* CBS:113178 | GU981570.1 | GU981646.1 | MN969287.1 | KF296450.1 |
| *Penicillium penarojense* CBS:113132 | GU981572.1 | GU981644.1 |  |  |
| *Penicillium elleniae* CBS:118136 | GU981611.1 | GU981664.1 |  |  |
| *Penicillium elleniae* CBS:118135 | GU981612.1 | GU981663.1 | MN969254.1 | KF296429.1 |
| *Penicillium elleniae* CBS:118134 | GU981610.1 | GU981662.1 |  |  |
| *Penicillium wotroi* CBS:118171 | GU981591.1 | GU981637.1 | MN969313.1 | KF296460.1 |
| *Penicillium wotroi* CBS:116295 | GU981589.1 | GU981635.1 |  |  |
| *Penicillium onobense* CBS:174.81 | MH861322.1 | GU981627.1 | MN969281.1 | KF296447.1 |
| *Penicillium araracuarense* CBS:113149 | GU981597.1 | GU981642.1 | MN969237.1 | KF296414.1 |
| *Penicillium araracuarense* CBS:113148 | GU981596.1 | GU981641.1 |  |  |
| *Aspergillus fumigatus* HGUP192001 | MZ541953.1 | OP312084.1 |  | MZ546146.1 |

Supplementary Table
